# Effects of emollient therapy with sunflower seed oil on neonatal growth and morbidity in Uttar Pradesh, India: a cluster-randomized, open-label, controlled trial

**DOI:** 10.1093/ajcn/nqab430

**Published:** 2022-01-04

**Authors:** Vishwajeet Kumar, Aarti Kumar, Shambhavi Mishra, Peiyi Kan, Sana Ashraf, Shambhavi Singh, Keona J H Blanks, Michael Baiocchi, Mika Limcaoco, Amit K Ghosh, Alok Kumar, Raghav Krishna, David K Stevenson, Lu Tian, Gary L Darmstadt, For the Shivgarh Emollient Research Group, Sana Ashraf, Gary L Darmstadt, Peter M Elias, Amit Kumar Ghosh, Peiyi Kan, Raghav Krishna, Aarti Kumar, Alok Kumar, Vishwajeet Kumar, Hina Mehrotra, Shambhavi Mishra, Pawankumar Patil, Arti Sahu, Pramod Singh, Shambhavi Singh, Vivek Singh, David K Stevenson, Lu Tian, Ranjana Yadav

**Affiliations:** Community Empowerment Lab, Lucknow, India; Community Empowerment Lab, Lucknow, India; Department of Statistics, Lucknow University, Lucknow, India; Prematurity Research Center, Department of Pediatrics, Stanford University School of Medicine, Stanford, CA, USA; Community Empowerment Lab, Lucknow, India; Community Empowerment Lab, Lucknow, India; Earth Systems Program, Stanford University, Stanford, CA, USA; Department of Epidemiology and Population Health, Stanford University School of Medicine, Stanford, CA, USA; Stanford Prevention Research Center, Stanford University School of Medicine, Stanford, CA, USA; Government of India, India; Government of Uttar Pradesh, India; Community Empowerment Lab, Lucknow, India; Prematurity Research Center, Department of Pediatrics, Stanford University School of Medicine, Stanford, CA, USA; Department of Health Research and Policy, Stanford University School of Medicine, Stanford, CA, USA; Prematurity Research Center, Department of Pediatrics, Stanford University School of Medicine, Stanford, CA, USA

**Keywords:** newborn growth, neonatal growth, neonatal health, neonatal morbidity, newborn morbidity, emollient, skin barrier

## Abstract

**Background:**

Newborn oil massage is a widespread practice. Vigorous massage with potentially harmful products and forced removal of vernix may disrupt skin barrier integrity. Hospitalized, very-preterm infants treated with sunflower seed oil (SSO) have demonstrated improved growth but community-based data on growth and health outcomes are lacking.

**Objectives:**

We aimed to test whether SSO therapy enhances neonatal growth and reduces morbidity at the population level.

**Methods:**

We conducted an open-label, controlled trial in rural Uttar Pradesh, India, randomly allocating 276 village clusters equally to comparison (usual care) and intervention comprised of promotion of improved massage practices exclusively with SSO, using intention-to-treat and per-protocol mixed-effects regression analysis.

**Results:**

We enrolled 13,478 and 13,109 newborn infants in demographically similar intervention and comparison arms, respectively. Adherence to exclusive SSO increased from 22.6% of intervention infants enrolled in the first study quartile to 37.2% in the last quartile. Intervention infants gained significantly more weight, by 0.94 g · kg^−1^ · d^−1^ (95% CI: 0.07, 1.82 g · kg^−1^ · d^−1^, *P* = 0.03), than comparison infants by intention-to-treat analysis. Restricted cubic spline regression revealed the largest benefits in weight gain (2–4 g · kg^−1^ · d^−1^) occurred in infants weighing <2000 g at birth. Weight gain in intervention infants was higher by 1.31 g · kg^−1^ · d^−1^ (95% CI: 0.17, 2.46 g · kg^−1^ · d^−1^; *P* = 0.02) by per-protocol analysis. Morbidities were similar by intention-to-treat analysis but in per-protocol analysis rates of hospitalization and of any illness were reduced by 36% (OR: 0.64; 95% CI: 0.44, 0.94; *P* = 0.02) and 44% (OR: 0.56; 95% CI: 0.40, 0.77; *P* < 0.001), respectively, in treated infants.

**Conclusions:**

SSO therapy improved neonatal growth, and reduced morbidities when applied exclusively, across the facility–community continuum of care at the population level. Further research is needed to improve demand for recommended therapy inside hospital as well as in community settings, and to confirm these results in other settings.

This trial was registered at www.isrctn.com as ISRCTN38965585 and http://ctri.nic.in as CTRI/2014/12/005282.

## Introduction

The epidermal barrier of the skin—the largest organ of the newborn infant—is developmentally compromised and easily injured at birth, especially in preterm infants, posing risks of accelerated water and heat loss, growth faltering, systemic infection, and mortality (1–5). High environmental pathogenic load in tropical settings, poor hygiene, and forceful, injurious removal of vernix at birth may all place newborn infants at risk (6–9).

Oil massage of newborns is a widespread practice throughout Africa, Asia, and the Mediterranean region (10–15). Application of high-linoleate sunflower seed oil (SSO) was found in mouse models of human infant skin to enhance skin barrier repair and integrity, whereas local products and oils routinely applied to newborns in high-mortality regions in South Asia—including mustard oil—and sub-Saharan Africa showed harmful effects (15–17). Mustard oil can have high concentrations of proinflammatory erucic acid, causing keratinocyte toxicity and skin and gastrointestinal tract inflammation (17, 18), and contamination with seeds of *Argemone mexicana* can cause epidemic dropsy (19). However, skin barrier integrity was similar in newborns massaged with SSO or mustard oil in the community in Nepal (20).

Before the advent of intralipids, topical applications of SSO, leading to absorption of fatty acids (21), were used in preterm infants to support growth and prevent or treat essential fatty acid deficiencies (22–26). Furthermore, linoleic acid (18:2n–6)—an essential fatty acid abundant in SSO—binds specifically to receptors on keratinocytes to accelerate and bolster skin development (27–29).

In low-resource settings, emollient therapy in preterm infants has been shown to increase fatty acid concentrations in blood (30, 31), improve thermoregulation (30, 32) and skin barrier condition and function (33–37), reduce the risk of serious infections (33–39) and mortality (39–41), enhance neurodevelopment (42–44), and improve growth (31, 35, 37, 39, 42, 44–54) during the neonatal period at high cost-effectiveness (55). Research on health benefits of emollient therapy in term infants is scarce, demonstrating transcutaneous absorption of lipids (56) and improvement in growth in 2 studies (44, 45) but not significantly in another (57).

Despite the risks due to compromised skin barrier function and the growing evidence for improved neonatal outcomes from emollient therapy, data are scarce regarding its utility as a public health strategy for improving newborn health. Previously we examined the impacts of gentle massage with SSO compared with usual skin care practices in a population-based cohort of newborn infants in a rural community in Uttar Pradesh, India. We showed no overall effect on neonatal mortality but a significant 52% reduction in mortality among the subgroup of very-low-birth-weight (VLBW) infants ≤1500 g (41). Here we examine the effects of SSO treatment on the neonatal growth and morbidity of the infants in that trial.

## Methods

### Study design

The study was a 2-arm, cluster-randomized, open-label, controlled public health intervention trial conducted in 276 contiguous village administrative units or clusters, each with an average population of ∼3000 in a rural community of 818,000 inhabitants of the Rae Bareli and Amethi districts of Uttar Pradesh. The intervention was aimed to modify pre-existing, nearly universal practices of newborn oil massage governed by social norms and conducted by traditional masseuses with defined service areas; thus, randomization was done at the cluster level. The primary outcome of the parent trial was neonatal mortality rate (NMR), which was reported separately (41). The sample size for the parent trial was calculated to enable measurement of a ≥15% reduction in NMR at a 5% level of significance with 90% power, as detailed previously (41, 58). Secondary outcomes included changes in oil massage practices, adherence to treatment, weight gain, and morbidities, and are the focus of this report.

### Ethical review and trial registration

The study received ethical clearance from an independent Institutional Ethics Committee at the Community Empowerment Lab and the Ethics Review Committee at the WHO, Geneva. The trial was registered at the ISRCTN (ISRCTN38965585) and the Clinical Trials Registry—India (CTRI) (CTRI/2014/12/005282) registries with WHO UTN # U1111-1158-4665. The trial protocol can be found at https://doi.org/10.7910/DVN/TGNC9H, Harvard Dataverse, V1.

### Randomization, allocation, and eligibility

Randomization was conducted at WHO Geneva to allocate 276 clusters equally to the 2 arms, as described in detail previously (41). Randomization was done at the cluster level to minimize contamination of the intervention into comparison clusters. Because allocation to the intervention or comparison arms could not be concealed from the implementation teams owing to the visible nature of the intervention, various measures were adopted to minimize contamination and observer bias. The intervention and evaluation teams were independent, with separate lines of management and no formal communication between them in order to ensure unbiased data collection. Data management protocols masked the cluster allocation from monitoring and analysis teams, which ensured that during execution of the study, data were never accessed or reported separately for the 2 study groups.

Following a community-consenting process with community leaders from each of the study clusters, a surveillance system was established for identifying pregnancies and births through 2-monthly cycles of door-to-door visitations, supported by a social network of key informants to notify project staff of births as early as possible. Pregnant women who planned to stay within their intervention cluster through the newborn period were provided informed consent and provisionally enrolled. All newborn infants identified in study clusters within 7 d of birth were eligible for inclusion in the study, enrolled after gaining informed consent from their mother, and analyzed as part of the cluster where they were first identified, irrespective of cross-migration. There were no prespecified exclusion criteria.

### Intervention and delivery strategy

Home visitations were the mainstay of intervention promotion and consisted of an antenatal interaction between intervention workers and families of pregnant women identified through demographic surveillance in their 25^th^ week of pregnancy. The first postnatal interaction was targeted for the first day (i.e., the day of birth) and the second postnatal visit was targeted for the seventh day after birth. The median time when the first postnatal visit occurred was day 2 (the day after birth) (IQR: days 1–3) for both intervention and comparison groups, as reported previously (41). Thus, the first-visit weight appears to be a good approximation for birth weight.

We promoted exclusive use of SSO as the emollient for massage during the neonatal period, including 3-times daily applications of ∼10 g SSO using gentle massage with washed hands. The usual practice of applying mustard oil 2–4 times daily, sometimes infused with herbs and often with vigorous massage (7–9), was not intervened upon in the comparison clusters. Infants in the intervention and comparison arms were treated with emollient an average of 2.7 and 2.4 times/d, respectively (41). The intervention delivery strategy was developed through a community-centric design process and trials of improved practices aimed to ensure early and exclusive applications of SSO.

The SSO supplied to families was cold-pressed and each batch of oil was quality tested in an accredited laboratory (SGS India Private Limited) to ensure high linoleic acid content (>60%) and purity from harmful chemicals. The oil was packaged into light-and-heat-protected sterile bottles with packaging that was designed through a participatory branding exercise and distributed under the label “*Saksham Sneh* [literally, “empowerment and maternal affection”] Newborn Baby Oil” to enhance its desirability and adoption.

Intervention workers had ≥12 y of education and were provided an initial 3-d classroom and 7-d on-the-job training on the intervention and behavior change management approach. They were each allocated an area covering an average of 1200 households. Intervention supervisors each covered 10–12 workers for mentoring, supervision, and resolution of ongoing field issues. A team of remote guidance assistants centrally scheduled and monitored intervention visitations through a call center.

Intervention workers received no training on newborn care and were instructed to not counsel families on other aspects of newborn care besides the intervention. Although washing of hands before SSO application was part of the recommended massage practices, overall benefits of handwashing and its practice beyond massage were not discussed with families. Recognizing that there were no corresponding visits in the comparison clusters which were comparable with the intervention worker visits, data collector visitations were scheduled on the same days as the intervention worker visits to mitigate any potential “Hawthorne effect” (59).

Intervention workers promoted but did not apply SSO; skin care practices remained under the management of families with support from traditional masseuses. During the antenatal interaction mothers received behavioral counseling on oil application and massage and a 100-mL bottle of SSO to facilitate early application as soon as possible after birth, whether the birth occurred at home or in a health facility. During the first postnatal interaction, intervention workers reinforced emollient practices and provided a 200-mL bottle of SSO for use during the remainder of the first week. The second postnatal interaction involved provision of the rest of the monthly supply of three 200-mL bottles of SSO and addressing any remaining queries of the families. Traditional masseuses (*n* = 1189) who serviced the intervention clusters were engaged to ensure that they acted as promoters of rather than barriers to intervention adoption and were trained and accredited on emollient application practices. In addition, monthly community meetings were conducted to reinforce the early and exclusive application of SSO with the recommended massage technique.

### Data collection and management

Data collectors had ≥12 y of education and received an initial 7-d classroom and 3-d on-the-job training on the questionnaires and their administration, pregnancy surveillance, use of electronic tablet devices, and weight measurement to the nearest 10 g using the American Weigh Scales AMW-SR-20 digital hanging scale. Each data collector was allocated an area covering ∼1000 households (4 clusters).

A baseline questionnaire for gathering data on socioeconomic status and pregnancy history was administered in the antenatal period during week 25 of gestation on the same day as the antenatal intervention visit. Data on handwashing, newborn oil massage practices (oil additives and use), morbidities (hospitalization, illness, skin infection, umbilical cord infection), and survival status were collected at the first visit (with the recall period from birth to the first postnatal visit), which occurred at a median of day 2 (IQR: days 1–3) (41); on the day 7 visit (with the recall period from the first to the second visit); and on the third visit targeted for day 29 (with the recall period from the second to the third visit). Data from the 3 visits were combined to generate measures over the neonatal period. Infants were weighed at the first visit and at the third visit.

Proprietary software on electronic tablet devices was utilized for data collection, and data were synchronized from the cloud to a MySQL database on a daily basis. The software included built-in checks for logical inconsistencies, skips, missing values, and range limits. Data collection home visitations were centrally scheduled and appointments were dispatched to data collectors’ tablet devices and monitored through a call center by a team of remote guidance assistants who also tracked the Global Positioning System locations of workers and data collection points. Quality checks consisted of in-the-field spot-checks and back-checks for 5% of all visits. All stillbirths and neonatal deaths were verified by a team of supervisors who were each responsible for 10 data collectors. Two data analysts who were blinded to group allocation reviewed the data quality regularly and provided interviewer- and data-specific feedback for improving data quality.

### Ethics and trial oversight

Consent was obtained from leaders in each of the study clusters, and written informed consent was obtained from the parents/guardians of all infants before enrollment. Procedures followed were in accordance with the Helsinki Declaration of 1975 as revised in 1983.

A group of expert child health clinical trialists from the WHO assisted in overseeing the conduct of the trial, coordinated the ethical review process at WHO Geneva, convened the Technical Advisory Group and the Data Safety Monitoring Board (DSMB), and coordinated the reporting of severe adverse events to the DSMB as described previously (41). The role of the DSMB in monitoring the trial based on mortality outcomes was described in detail previously (41).

### Statistical analysis

Analyses were done in SAS version 9.0 (SAS) and replicated in STATA version 13.0 (StataCorp LLC) for verification.

### Adherence to treatment

Analysis of adherence to exclusive use of emollient (SSO in the intervention arm, mustard oil in the comparison arm) as a measure of treatment fidelity utilized individual-level data without adjustment for either cluster-size variation or covariates.

### Intention-to-treat analysis

We had intended to compare cluster-level measures of changes in practices (addition of *bukwa* to oil, handwashing), growth (g · kg^−1^ · d^−1^, g/kg), and morbidities (hospitalization, illness, skin infection, umbilical cord infection) across groups using a 2-sample *t* test. Owing to wide variation in cluster sizes we applied a more statistically efficient method of individual-level analysis comparing infants randomly assigned to the intervention group with infants randomly assigned to the comparison group, adjusting for within-cluster variations using mixed-effects regression [linear for continuous outcome (growth) or logistic for binary outcome (practices, morbidities) as appropriate] with group (intervention and comparison group) as a fixed effect and cluster as a random effect (58), as described previously (41). Given the large sample size and randomization, we present crude estimates for intention-to-treat analyses without adjusting for any baseline covariates except that the SE of the parameter estimates was adjusted for within-cluster variations via random-effects modeling. As a sensitivity analysis, multivariate mixed-effects regression analyses were repeated with adjustment for selected covariates. Specifically, caste, first-visit weight (as a proxy for birth weight), delivery attendant, gravidity, maternal age, maternal education, sex of the infant, and multiple births were included in logistic regression analyses of practices and morbidities; sensitivity linear regression analysis for growth, expressed as g · kg^−1^ · d^−1^or g/kg, did not include first-visit weight as a covariate. Intention-to-treat mixed-effects regression analyses which were adjusted for covariates are presented in **Supplemental Tables 1**and**2**. In the analysis for growth, we examined the potentially heterogenous treatment effect according to the first-visit weight and the analyses were adjusted for the same set of covariates as aforementioned (except for first-visit weight), because the effective sample size was smaller for subgroups of infants with specific first-visit weights and there was a chance of imperfect randomization. The outcome for the analysis for growth was the average weight gain per day per first-visit weight and the association between the first-visit weight and outcome was modeled via a nonparametric restricted cubic spline regression with 4 preselected knots within each study group (60). ORs and 95% CIs for practices and morbidities were calculated using mixed-effects logistic regression, with comparison infants as the reference. *P* values for the morbidity analysis were also adjusted using the linear step-up, false discovery rate (FDR) controlling procedure of Benjamini and Hochberg (61) and the results are reported in the text. Type I error, α, was controlled at 0.05.

### Per-protocol analysis

To further assess the impacts of SSO therapy, we conducted per-protocol analysis of data on growth and morbidities using data on adherence to treatment. The follow-up period was from birth (day 1) to death or 28 completed days after birth. Complete adherence to SSO was defined as using SSO to initially cleanse the newborn infant, applying SSO at the first application during the first 6 h after birth, and subsequently applying SSO exclusively during the entire follow-up period. An analogous definition was used to identify newborn infants who were treated exclusively with mustard oil. Infants treated with additional regimens (e.g., >1 oil) were not included owing to the biased association of increased likelihood of alternative regimens with increased survival time and potential for morbidities. We compared growth and morbidities of infants who had been randomly assigned to the intervention group and whose caregivers strictly adhered to the exclusive use of SSO (“exclusive SSO”) with those in the comparison group who received massage with mustard oil exclusively (“exclusive mustard oil”) using the same regression methods as for the intention-to-treat analysis, i.e., individual-level analysis accounting for within-cluster variations using mixed-effects regression. Given that a subset of infants in the intervention and comparison groups were selected for these analyses based on treatment adherence criteria rather than randomized group assignment, we present estimates that are adjusted for covariates. Crude estimates of growth and morbidity effects based on per-protocol analyses unadjusted for covariates are shown in Supplemental Tables 1 and 2.

## Results

### Study population

**FIGURE 1:**
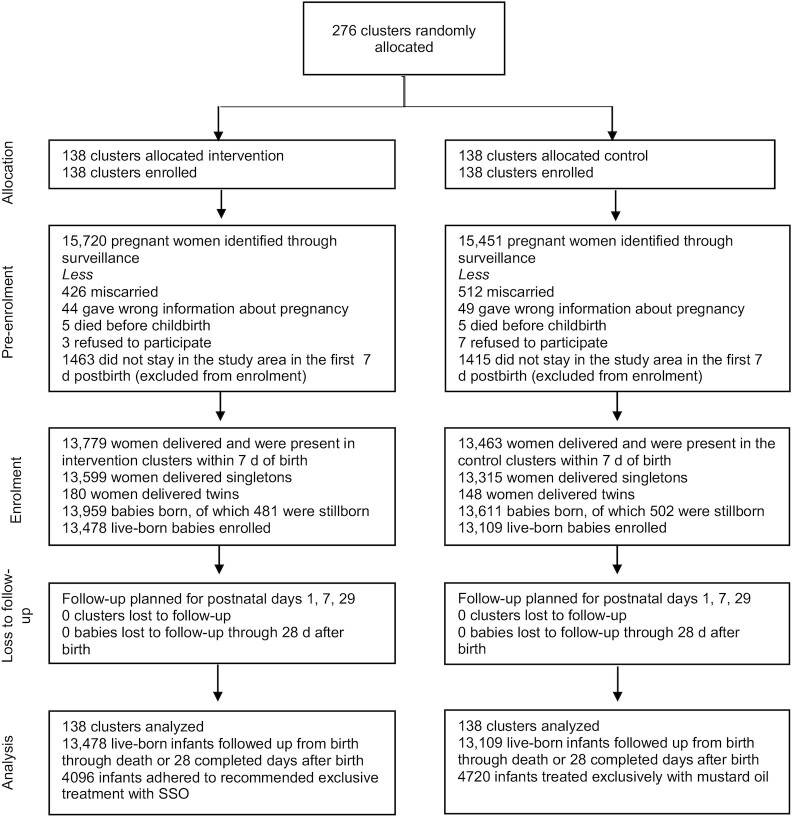
Consolidated Standards of Reporting Trials (CONSORT) diagram for a cluster-randomized, open-label, controlled trial of impact of emollient therapy with sunflower seed oil on growth and morbidity of neonatal infants in a population-based cohort in Uttar Pradesh, India. Adapted from Kumar et al. (41). SSO, sunflower seed oil.

A total of 13,478 live-born infants in the intervention clusters and 13,109 infants in the comparison clusters were followed up from birth to death or 28 completed days during November 2014–October 2016 for measures of weight and morbidity (**Figure 1**). Among infants randomly assigned to the intervention arm, 4096 infants (30.4%) received SSO therapy exclusively, whereas 4720 infants (36.0%) who were randomly assigned to the comparison arm received mustard oil exclusively.

Infants randomly assigned to the 2 study groups were comparable in baseline characteristics (**Table 1**) (41). Briefly, mothers had a mean age of 25.4 y, most (86.7%) were Hindu and about one-third (35.9%) were from scheduled castes, one-third (33.3%) were illiterate, and most (84.8%) gave birth in a health care facility with a skilled birth attendant (83.1%). Characteristics were also similar for the participants in the intervention arm who adhered to exclusive use of SSO and those in the comparison arm who practiced exclusive use of mustard oil (Table 1).

**FIGURE 2:**
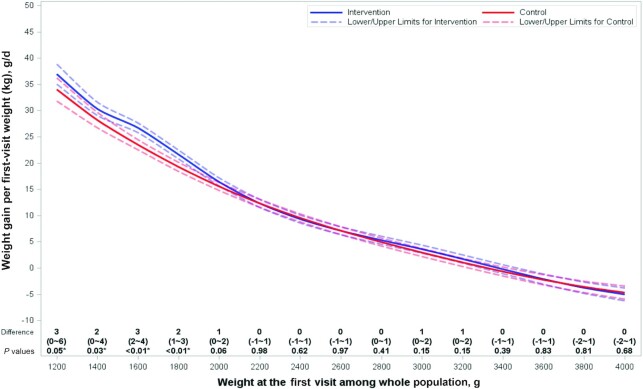
Neonatal weight gain (g · kg^−1^ · d^−1^) as a function of first-visit weight (a proxy for birth weight) in infants in intervention and comparison clusters in Uttar Pradesh, India, modeled via a nonparametric restricted cubic spline regression with 4 preselected knots within each study group, adjusted for covariates as described in the Methods. *n* = 11,118, intervention; *n* = 10,834, comparison. *Indicates a statistically significant difference in weight gain between infants in the intervention vs. comparison clusters.

**TABLE 1:** Baseline characteristics of the study population randomly assigned to intervention and comparison groups for intention-to-treat analysis and included in per-protocol analysis^1^

	Intention-to-treat		Per-protocol	
Characteristic	Comparison	Intervention	Comparison, exclusive mustard oil	Intervention, exclusive sunflower seed oil
Households per cluster, median *n* (range)	458 (208–2286)	477 (210–3061)		
Total live births	13,109 (49.3)	13,478 (50.7)	4720 (53.5)	4096 (46.5)
Singleton	12,840 (97.9)	13,146 (97.5)	4617 (97.8)	3994 (97.5)
Multiple	269 (2.1)	332 (2.5)	103 (2.2)	102 (2.5)
Male	6820 (52.0)	7042 (52.3)	2431 (51.5)	2114 (51.6)
Religion				
Hindu	11,346 (86.6)	11,675 (86.6)	4185 (88.7)	3502 (85.5)
Muslim	1743 (13.3)	1787 (13.3)	532 (11.3)	588 (14.4)
Other	14 (0.1)	13 (0.1)	3 (0.1)	6 (0.1)
Maternal age, y	25.3 ± 3.7	25.4 ± 3.7	25.4 ± 3.8	25.5 ± 3.7
Caste				
General	2038 (15.6)	2001 (14.9)	676 (14.3)	560 (13.7)
Other backward caste	6372 (48.6)	6622 (49.1)	2165 (45.9)	2003 (48.9)
Scheduled caste/scheduled tribe	4693 (35.8)	4852 (36.0)	1879 (39.8)	1533 (37.4)
Maternal education				
Illiterate	4324 (33.0)	4531 (33.6)	1536 (32.5)	1416 (34.6)
Primary completed	2913 (22.2)	3215 (23.9)	1050 (22.2)	949 (23.2)
Tenth grade completed	3843 (29.3)	3720 (27.6)	1451 (30.7)	1168 (28.5)
Secondary and above completed	2023 (15.4)	2009 (14.9)	683 (14.5)	563 (13.7)
Delivery place				
Health facilities	11,038 (84.2)	11,497 (85.3)	3878 (82.2)	3558 (86.8)
On the way to a facility from home	40 (0.3)	30 (0.2)	19 (0.4)	7 (0.2)
Home	2031 (15.5)	1949 (14.5)	823 (17.4)	531 (13.0)
Delivery attendant				
Physician	4059 (31.0)	4931 (36.6)	1420 (30.1)	1228 (30.0)
Auxiliary Nurse Midwife/staff nurse	6710 (51.2)	6397 (47.5)	2374 (50.3)	2273 (55.5)
Others	2340 (17.8)	2148 (15.9)	926 (19.6)	595 (14.5)
Delivery type				
Normal	12,136 (92.6)	12,407 (92.1)	4396 (93.1)	3891 (95.0)
Assisted (forceps) or episiotomy	670 (5.1)	704 (5.2)	201 (4.5)	135 (3.3)
Cesarean	303 (2.3)	325 (2.4)	113 (2.4)	70 (1.7)
Gravidity				
1	5105 (39.0)	5282 (39.2)	1841 (39.0)	1594 (38.9)
2–3	5499 (41.9)	5470 (40.6)	1972 (41.8)	1660 (40.5)
≥4	2499 (19.1)	2723 (20.2)	907 (19.2)	842 (20.6)
Toilet type				
Open defecation	11,718 (89.4)	12,203 (90.6)	4223 (89.5)	3767 (92.0)
Latrine/toilet	1385 (10.6)	1272 (9.4)	497 (10.5)	329 (8.0)
First-visit weight, g	2607 ± 509	2575 ± 521	2570.96 ± 530.5	2575.67 ± 493.01
Age at measurement of first-visit weight, d	2.6 ± 1.5	2.6 ± 1.5	2.6 ± 1.6	2.5 ± 1.5

1 Adapted from Kumar et al. (41). Values are *n* (%) or mean ± SD unless indicated otherwise.

### Adherence to treatment

Over the course of implementation, increasing proportions of infants in the intervention clusters were treated exclusively with SSO as recommended (**Table 2**). Adherence to exclusive SSO increased from 22.6% of infants enrolled in the first quartile to 37.2% of infants enrolled in the last quartile. In contrast, exclusive use of mustard oil for massage of newborn infants in the comparison clusters showed less change, ranging from 32.4% in the first quartile of the study to 38.3% in the final quartile.

**TABLE 2:** Adherence to emollient treatment^1^

Time period (quartiles of enrollment)	Infants enrolled in emollient arm, *n*	Infants in emollient arm treated exclusively with SSO, *n*	Exclusive SSO in emollient arm,^2^ %	Infants enrolled in comparison arm, *n*	Infants in comparison arm treated exclusively with MO, *n*	Exclusive MO in comparison arm,^3^ %
11/10/2014–09/26/2015	3324	750	22.6	3346	1083	32.4
09/27/2015–02/17/2016	3376	849	25.2	3259	1095	33.6
02/18/2016–07/10/2016	3418	1248	36.5	3220	1285	39.9
07/11/2016–10/15/2016	3360	1249	37.2	3284	1257	38.3
Total	13,478	4096	30.4	13,109	4720	36.0

1 Values are proportions of infants in the intervention clusters treated exclusively with SSO and infants in the comparison clusters who received exclusive applications of MO as a percentage of the infants enrolled by quartile periods of enrollment, unless indicated otherwise. MO, mustard oil; SSO, sunflower seed oil.

2 Exclusive SSO was defined as use of SSO to initially cleanse the newborn infant, applying SSO at the first application during the first 6 h after delivery, and subsequently applying SSO exclusively during the entire follow-up period.

3 Exclusive MO was defined as use of MO to initially cleanse the newborn infant, applying MO at the first application during the first 6 h after delivery, and subsequently applying MO exclusively during the entire follow-up period.

### Intention-to-treat analysis

#### Practices

About 28% of infants in comparison clusters were massaged with *bukwa*, a mixture of oil and ground-up grains infused with herbs (**Table 3**). This potentially harmful practice was reduced to ∼6% of infants in the intervention clusters, a significant 88% (OR: 0.12; 95% CI: 0.08, 0.18; *P* < 0.0001) reduction. The odds of handwashing before caring for the infant, including oil application, were increased by 78% over the neonatal period (OR: 1.78; 95% CI: 1.01, 3.13; *P* < 0.0001).

**TABLE 3:** Practices promoted in the intervention clusters which were reported for infants in intervention and comparison clusters during the neonatal period from birth through the third (day 29) postnatal visit

Practice	Comparison *n* [% of total − (missing + died)] (total *n* = 13,109)	Intervention *n* [% of total − (missing + died)] (total *n* = 13,478)	OR^1^ (95% CI)	*P* value
Addition of *bukwa*			0.12 (0.08, 0.18)	<0.0001
Yes	3457 (28.4)	798 (6.4)		
No	8696 (71.6)	11,621 (93.6)		
Died before visit	732	740		
Missing	224	319		
Handwashing before caring for the newborn (including oil application)			1.78 (1.01, 3.13)	<0.0001
Yes	3766 (33.5)	5256 (46.1)		
No	7466 (66.5)	6135 (53.9)		
Died before visit	732	740		
Missing	1145	1347		

1 Individual-level analysis showing crude estimates accounting for within-cluster variations using mixed-effects logistic regression. Additional adjustment for covariates (caste, first-visit weight, delivery attendant, gravidity, maternal age, maternal education, sex of the infant, multiple births) produced similar results for addition of *bukwa* (adjusted OR: 0.12; 95% CI: 0.09, 0.18; *P* < 0.0001) and for handwashing (adjusted OR: 1.81; 95% CI: 1.03, 3.19; *P* < 0.0001).

#### Growth

Significantly higher weight gain velocity by a difference of 0.94 g · kg^−1^ · d^−1^ (95% CI: 0.07, 1.82 g · kg^−1^ · d^−1^; *P* = 0.03) was found among infants in the intervention clusters, who gained 13.60 g · kg^−1^ · d^−1^ (95% CI: 12.98, 14.22 g · kg^−1^ · d^−1^), whereas infants in the comparison clusters gained 12.65 g · kg^−1^ · d^−1^ (95% CI: 12.04, 13.27 g · kg^−1^ · d^−1^), over the neonatal period (**Table 4**). Exploratory analysis by sex revealed similar results for emollient-treated male and female infants who gained a mean of 0.92 g · kg^−1^ · d^−1^ (95% CI: −0.01, 1.85 g · kg^−1^ · d^−1^; *P* = 0.05) and 1.00 g · kg^−1^ · d^−1^ (95% CI: 0.04, 1.96 g · kg^−1^ · d^−1^; *P* = 0.04) more weight, respectively, than infants in the comparison group. The difference in weight gain velocity between SSO-treated and comparison infants reached a mean of 4.21 g · kg^−1^ · d^−1^ (95% CI: −3.44, 11.87 g · kg^−1^ · d^−1^) in infants born weighing ≤1500 g. The difference in weight gain over the neonatal period was also significantly greater, by 28.43 g/kg (95% CI: −0.46, 57.32 g/kg; *P* = 0.05), among infants in the intervention (400.82 g/kg; 95% CI: 380.39, 421.24 g/kg) than in the comparison (372.39 g/kg; 95% CI: 351.95, 392.82 g/kg) group (Table 4). Similar results were found for covariate-adjusted analyses (Supplemental Table 1). Spline analysis revealed significantly higher weight gain velocity in infants in the intervention than in the comparison group, which was accentuated to ∼2–4 g · kg^−1^ · d^−1^ for infants who had a first-visit weight below ∼2000 g (**Figure 2**).

**TABLE 4:** Mean differences in weight gain over the neonatal period between intervention and comparison clusters of infants by intention-to-treat analysis, and infants in the intervention group treated exclusively with SSO compared with infants in the comparison group massaged exclusively with MO by per-protocol analysis^1^

	Comparison	Intervention	Mean difference
Intention-to-treat analysis^2^			
Weight gain daily per first-visit weight, g · kg^−1^ · d^−1^ (95% CI)	12.65 (12.04, 13.27)	13.60 (12.98, 14.22)	0.94 (0.07, 1.82), *P* = 0.03
Weight gain per first-visit weight, g/kg (95% CI)	372.39 (351.95, 392.82)	400.82 (380.39, 421.24)	28.43 (−0.46, 57.32), *P* = 0.05
Per-protocol analysis^3^			
Weight gain daily per first-visit weight, g · kg^−1^ · d^−1^	13.29 (12.01, 14.58)	14.61 (13.32, 15.90)	1.31 (0.17, 2.46), *P* = 0.02
Weight gain per first-visit weight, g/kg	393.80 (357.60, 430.00)	426.04 (389.69, 462.39)	32.24 (3.13, 61.34), *P* = 0.03

1 MO, mustard oil; SSO, sunflower seed oil.

2 Individual-level analysis showing crude estimates accounting for within-cluster variations using mixed-effects linear regression. Infants who died or for whom weight data were missing were excluded from analysis. Comparison, *n* = 10,834; intervention, *n* = 11,118.

3 Individual-level analysis showing adjusted estimates accounting for within-cluster variations using mixed-effects linear regression and adjusting for covariates (caste, delivery attendant, gravidity, maternal age, maternal education, sex of the infant, and multiple births). Infants who died or for whom weight or covariate data were missing were excluded from analysis. Comparison: exclusive MO, *n* = 3782; intervention: exclusive SSO, *n* = 3534.

#### Morbidity

Across both the intervention and comparison groups, ∼2.5% of infants were hospitalized and 11%–12% developed any illness during the neonatal period, by care-giver self-report (**Table 5**). Skin infections were reported in 3%–4% of infants and umbilical cord infections occurred in 8%–9%. There were no significant differences in measures of morbidity by care-giver self-report between infants in the intervention and comparison groups (Table 5).

**TABLE 5:** Infant morbidity in the neonatal period in intervention and comparison clusters^1^

Morbidity outcomes	Total, *n*	No, *n*	Yes, *n* (% of total)	OR^2^	*P* value^2^
Hospitalization^3^					
Intention-to-treat					
Intervention	13,478	13,139	339 (2.5)	0.99 (0.79, 1.23)	0.901
Comparison	13,109	12,789	320 (2.4)		
Per-protocol					
Exclusive SSO in intervention	4096	4021	75 (1.8)	0.64 (0.44, 0.94)	0.022
Exclusive MO in comparison	4720	4584	136 (2.9)		
Any illness^4^					
Intention-to-treat					
Intervention	12,449	11,103	1346 (10.8)	0.85 (0.67, 1.07)	0.176
Comparison	12,180	10,657	1523 (12.5)		
Per-protocol					
Exclusive SSO in intervention	3838	3576	262 (6.8)	0.56 (0.40, 0.77)	<0.001
Exclusive MO in comparison	4222	3781	441 (10.4)		
Skin infection^5^					
Intention-to-treat					
Intervention	13,478	12,966	512 (3.8)	1.16 (0.81, 1.64)	0.416
Comparison	13,109	12,630	479 (3.6)		
Per-protocol					
Exclusive SSO in intervention	4096	3987	109 (2.7)	0.92 (0.56, 1.54)	0.764
Exclusive MO in comparison	4720	4553	167 (3.5)		
Umbilical disorder^6^					
Intention-to-treat					
Intervention	12,443	11,359	1084 (8.7)	1.02 (0.73, 1.42)	0.902
Comparison	12,188	11,146	1042 (8.5)		
Per-protocol					
Exclusive SSO in intervention	3832	3600	232 (6.1)	0.68 (0.45, 1.04)	0.073
Exclusive MO in comparison	4218	3914	304 (7.2)		

1 MO, mustard oil; SSO, sunflower seed oil.

2 For intention-to-treat models, crude estimates are shown from individual-level mixed-effects logistic regression analysis accounting for within-cluster variations. For per-protocol models, adjusted estimates are shown from individual-level mixed-effects logistic regression analysis accounting for within-cluster variations and adjusting for covariates (caste, first-visit weight, delivery attendant, gravidity, maternal age, maternal education, sex of the infant, and multiple births).

3 “Was the baby ever hospitalized?”

4 “Did the baby ever suffer from any health problem?”

5 “Did the baby ever have any boil on the skin with pus in it?”

6 “Did the baby ever suffer from a cord problem?”

### Per-protocol analysis

#### Growth

Weight gain velocity was significantly higher, by a mean difference of 1.31 g · kg^−1^ · d^−1^ (95% CI: 0.17, 2.46 g · kg^−1^ · d^−1^; *P* = 0.02), in infants in the intervention group who were treated exclusively with SSO (14.61 g · kg^−1^ · d^−1^; 95% CI: 13.32, 15.90 g · kg^−1^ · d^−1^) than in infants in the comparison group who were massaged exclusively with mustard oil (13.29 g · kg^−1^ · d^−1^; 95% CI: 12.01, 14.58 g · kg^−1^ · d^−1^) (Table 4). Exploratory analysis by sex found that the difference in weight gain velocity in intervention infants exclusively treated with SSO compared with comparison infants exclusively treated with mustard oil was significantly higher for females (1.49 g · kg^−1^ · d^−1^; 95% CI: 0.18, 2.80 g · kg^−1^ · d^−1^; *P* = 0.03) and showed a trend in males (1.20 g · kg^−1^ · d^−1^; 95% CI: −0.08, 2.48 g · kg^−1^ · d^−1^; *P* = 0.07). SSO-treated infants showed greater weight gain by 32.24 g/kg (95% CI: 3.13, 61.34 g/kg; *P* = 0.03) over the neonatal period than mustard oil–treated infants. Similar results were found for crude estimates unadjusted for covariates (Supplemental Table 1).

#### Morbidity

Rates of morbidities among infants in the intervention clusters who were treated exclusively with SSO as recommended (i.e., per protocol) were generally lower than among infants in the intervention clusters (i.e., intention to treat) and than among the infants in the intervention clusters who did not adhere to the recommended therapy with SSO (Table 5). For example, 1.8% (75 of 4096) of intervention infants who were treated per-protocol with SSO, 2.5% (339 of 13,478) of all infants in intervention clusters, and 2.8% (339 − 75/13478 − 4096) of infants in intervention clusters who were not treated per-protocol were hospitalized. Similarly, 6.8% of intervention infants treated exclusively with SSO, compared with 10.8% of all infants in intervention clusters and 12.6% of infants in intervention clusters who did not receive exclusive SSO, developed any illness during the neonatal period (Table 5). Similar beneficial patterns were seen for these same intervention subgroups for skin infections (2.7%, 3.8%, and 4.3%, respectively) and umbilical cord disorders (6.1%, 8.7%, and 9.9%, respectively). In contrast, there were little differences in rates of morbidities for infants in the comparison clusters who were massaged exclusively with mustard oil compared with all infants in the comparison clusters or with infants in comparison clusters who were not treated exclusively with mustard oil (Table 5). For example, among infants in the comparison group, hospitalization was reported in 2.9% of infants who received oil massage exclusively with mustard oil, 2.4% (1082 of 6876) of all infants in the comparison group, and 2.2% of infants who did not receive mustard oil exclusively. Any illness was reported in 10.4% of infants in the comparison groups who received oil massage exclusively with mustard oil, 10.8% of all infants in the comparison group, and 13.6% of infants who did not receive mustard oil exclusively. Weaker patterns were also seen for these same comparison subgroups for skin infections (3.5%, 3.6%, and 3.7%, respectively) and umbilical cord disorders (7.2%, 8.5%, and 9.3%, respectively).

Significant reductions by per-protocol regression analysis were found among infants in the intervention clusters who were treated exclusively with SSO compared with infants in the comparison clusters who were massaged exclusively with mustard oil in rates of hospitalization (OR: 0.64; 95% CI: 0.44, 0.94; *P* = 0.022) and any illness (OR: 0.56; 95% CI: 0.40, 0.77; *P* < 0.001) (Table 5). A trend was found for reduction in umbilical disorders (OR: 0.68; 95% CI: 0.45, 1.04; *P* = 0.073) and no significant difference was found for skin infections. Similar results were found for crude estimates unadjusted for covariates (Supplemental Table 2). After multiple-test adjustment, the FDR *P* value for hospitalization was 0.11 and any illness remained statistically significant with a FDR *P* value of 0.005.

There were no severe adverse events or unintended effects, as reported previously (41).

## Discussion

To our knowledge, this is the first study to examine population-level impacts of topical emollient therapy along the facility–community continuum of care in which treatment was initiated as soon as possible for all facility- and home-born infants. Previous studies of emollient therapy impacts on newborn growth have been initiated in hospital settings (23–26, 31, 35, 37, 39, 42, 44–54, 57, 62), although in some cases infants continued treatment and were followed up at home after discharge (35, 46, 47, 52, 62), and with few exceptions (45, 44, 57) prior studies have focused on preterm infants; studies have not reported on morbidities. The comparison group in prior studies typically received no skin care applications in a controlled hospital environment, whereas our comparison was usual skin care practices in the community, most notably mustard oil massage, a near-universal practice throughout South Asia (10–13). Standard of care incorporated an abrasive mixture of oil and pulverized grains (i.e., *bukwa*), which was rubbed on the skin of about one-quarter of infants in our comparison group. Finally, SSO massage was administered by families and not by intervention workers. Therefore, our study provides estimates of the effectiveness of SSO therapy in improving population-level newborn health.

We found that intervention with SSO therapy significantly improved the growth of newborn infants compared with usual practices by ∼1 g · kg^−1^ · d^−1^, which is below that reported previously (31, 35, 37, 39, 42, 44–54) and summarized in meta-analyses (1.1–2.9 g · kg^−1^ · d^−1^) (37, 39, 53, 54) for hospitalized, very-preterm infants under efficacy conditions. Although acceptance of SSO (i.e., any use) was high in intervention infants (89%) (41), because adherence to exclusive use of SSO was low in our study—increasing by 65% from 22.6% (first quartile) to 37.2% (fourth quartile) of infants in the intervention group—per-protocol analysis was important. Per-protocol analysis revealed that infants in the intervention group who were exclusively treated with SSO as intended showed a 1.3-g · kg^−1^ · d^−1^ greater growth velocity than infants in the comparison group who exclusively received mustard oil massage. The increase compared with intention-to-treat analysis was small (∼0.4 g · kg^−1^ · d^−1^), which may imply little dose response; however, the total dose of SSO was not precisely quantified and thus dose response requires further research. Spline analysis further indicated that the magnitude of growth promotion was highest among infants weighing <2000 g at birth, reaching ∼2–4 g · kg^−1^ · d^−1^, and regression analysis revealed greater weight gain by ∼4 g · kg^−1^ · d^−1^ in intervention than in comparison infants who were born weighing ≤1500 g. This magnitude of weight gain is comparable with that for infants born weighing <2000 g provided Kangaroo Mother Care (KMC) (4 g · kg^−1^ · d^−1^) (63).

As part of this same trial, we found that mortality risk was reduced by 52% in the subgroup of highly vulnerable VLBW infants (≤1500 g) and by per-protocol analysis mortality risk was reduced by 58% for infants (regardless of birth weight) in the intervention arm who received exclusive SSO treatments compared with infants in the comparison arm who received mustard oil massage exclusively (41). We attempted to conduct a post hoc analysis of the connection between neonatal weight gain and mortality using residual inclusion modeling to isolate variation in the outcome of mortality (64). Data on growth—which were limited to first-visit weight and weight at the third (day 29) visit and were missing for most deaths—were insufficient, however, to enable such an analysis. We found no prior reports quantifying the relation between change in growth in the neonatal period and mortality risk, but before the introduction of WHO growth standards for children in 2006–2009, studies generally showed an exponential increase in risk of child death with anthropometric *z* scores below −2, as well as increased risk of child death after periods of growth deceleration (65–69). More recently, with the availability of WHO standards for child growth velocity, analysis of longitudinal growth monitoring data from a cohort of children aged 3–24 mo in the Democratic Republic of Congo demonstrated that a weight velocity *z* score of −3 was associated with a 7.9-fold increase in RR of mortality in the subsequent 3-mo period (70). Young age (e.g., <1 y) was the strongest predictor for mortality risk (71) and growth velocity *z* scores reflecting recent weight loss were particularly useful in predicting death in the short term (i.e., within 3 mo).

Risk of neonatal mortality is greatest in the first week after birth, owing to a complex, often interacting mix of direct and indirect causes (72). Although nutrition of the mother and the newborn modifies the risk of various causes of neonatal mortality (73), susceptibility to mortality is in part independent of nutritional status; moreover, anthropometry incompletely characterizes newborn nutritional status (71, 74). However, body composition of the newborn infant (e.g., high skin surface area and low muscle mass compared with total body weight) may compound the effects of undernutrition, resulting in higher vital risk in association with poor growth than in older children. Muscle energy and protein reserves are a smaller proportion of total body weight, and fluid and heat losses are exponentially higher with increasing degrees of prematurity and potentially with undernutrition too, and thus may reach critical levels more easily (1, 4, 75, 76).

Although one would predict that growth promotion from emollient therapy would be associated with reduced risk of mortality, empirical evidence that causally connects a change in child growth with a change in the probability of mortality is lacking. Future residual inclusion modeling could offer suggestive causal evidence, but would require study design improvements, including *1*) sufficient power to detect a mortality effect; *2*) collection of intermediate growth data in addition to birth weight and day 29 weight measures; *3*) collection of weight, when possible, at the time of death; and *4*) additional information about other causal pathways from the treatment to the outcomes of interest (i.e., to mitigate challenges to the exclusion restriction assumption).

Further research is needed on potential approaches to improving adherence to recommended therapy, particularly in community settings. In this study, intervention workers had only 3 communication-based interactions aimed to enable families to initiate, adhere to, and sustain the therapy. Although this optimized feasibility and scalability, the community's inherent belief in the goodness of mustard oil appeared to be strong, suggesting that more intensive behavior change management is required to shift deeply entrenched community norms (8, 9, 77). Further research is also warranted on interactions of emollients, massage technique, and environmental conditions on skin barrier function in newborn infants (20, 78).

The mechanism of growth promotion by topical treatment with SSO likely occurred through a combination of local and systemic effects, including improved barrier integrity, reduced transepidermal water and heat loss, enhanced innate antimicrobial barrier defense, and reduced pathogen entry and immune activation, thus preserving energy (36, 38, 79–81). In addition, linoleic acid binds to peroxisome proliferator–activated receptor α receptors on keratinocytes to accelerate skin development, which is particularly important for preterm and undernourished infants (28, 29). Absorption of fatty acids provides building blocks and energy for growth (21, 22, 30, 31, 56), and emollient therapy may modulate the skin microbiome, as seen in young children with severe acute malnutrition in Bangladesh (82, 83).

This study had several limitations. Intervention workers could not be blinded to treatment group allocation; however, data collection workers had no information on group assignment. The study was not designed to distinguish the impacts of improved oil as opposed to improved application practices. Limitations in adherence to recommended treatment may have limited the impact of the intervention. Finally, limited data on growth ultimately precluded causal insight into the relation between growth and mortality.

In conclusion, considering the population-level reduction in mortality (41) along with increased growth of the subgroup of VLBW infants (41), there is an emerging evidence base for promotion of improved emollient therapy in the most highly vulnerable VLBW infants along the facility–community continuum of care. Research on integration of emollient therapy with KMC in infants born weighing <2000 g in facility and community settings is recommended (84). Treatment at the population level is not currently recommended. Further research is warranted to develop innovative approaches to improving adherence to recommended practices, and to exploring improvements in emollient composition (16, 17, 85), to optimize health benefits of emollient therapy for all newborn infants, including in sub-Saharan Africa where data are scarce.

## Supplementary Material

nqab430_Supplemental_File

## Data Availability

Data described in the article, code book, and analytic code will be made publicly and freely available without restriction at https://stanfordmedicine.app.box.com/folder/127704102699. The trial protocol can be found at Harvard Dataverse, V1, https://doi.org/10.7910/DVN/TGNC9H.

## Notes

Supported by a grant to the Community Empowerment Lab (to VK) from the WHO through grant INV-007723 from the Bill & Melinda Gates Foundation.

Views expressed are those of the authors and are not necessarily those of the WHO or the Bill & Melinda Gates Foundation.

Supplemental Tables 1 and 2 are available from the “Supplementary data” link in the online posting of the article and from the same link in the online table of contents at https://academic.oup.com/ajcn/.

Abbreviations used: DSMB, Data Safety Monitoring Board; FDR, false discovery rate; KMC, Kangaroo Mother Care; NMR, neonatal mortality rate; SSO, sunflower seed oil; VLBW, very-low-birth-weight.
