# Supplementary material for: Effects of emollient therapy with sunflower seed oil on neonatal growth and morbidity in Uttar Pradesh, India: a cluster-randomized, open-label, controlled trial
Source: Am J Clin Nutr. 2022 Jan 4;115(4):1092–104. doi: 10.1093/ajcn/nqab430 (PMC8970981; doi:10.1093/ajcn/nqab430)
Supplement: nqab430_Supplemental_File [file nqab430_supplemental_file.docx]

**On-line Supplemental Material**

**“Effects of emollient therapy with sunflower seed oil on neonatal growth and morbidity in Uttar Pradesh, India: a cluster-randomized, open-label, controlled trial**

**First author: Vishwajeet Kumar; corresponding author: Gary L. Darmstadt**

**Supplemental Table 1.** Mean differences [with 95% confidence intervals (CIs)] in

weight gain over the neonatal period between intervention and comparison clusters

of infants by intention-to-treat analysis, and in infants in the intervention group treated

exclusively with sunflower seed oil (SSO) compared to infants in the comparison group

massaged exclusively with mustard oil (MO) in per-protocol analysis.

| **Intention-to-treat analysis^1^** | | |
| --- | --- | --- |
| **Comparison** | **Intervention** | **Mean difference (95% CI), p-value** |
| **Weight gain daily per first visit weight: g/kg/d** | | |
| 12.85 (12.02, 13.67) | - 1. (12.97, 14.62) | 0.95 (0.07, 1.82), p=0.03 |
| **Weight gain per first visit weight: g/kg** | | |
| 377.79 (352.10, 403.48) | 406.12 (380.38, 431.86) | 28.33 (-0.26, 56.91), p=0.05 |
| **Per protocol analysis^2^** | | |
| **Comparison:**  **Exclusive MO** | **Intervention:**  **Exclusive SSO** | **Mean Difference (95% CI), p-value** |
| **Weight gain daily per first visit weight: g/kg/d** | | |
| 12.63 (11.81-13.45) | 13.90 (13.10-14.70) | 1.28 (0.13-2.42), p=0.03 |
| **Weight gain per first visit weight: g/kg** | | |
| 371.09 (347.39-394.79) | 401.76 (378.59-424.93) | 30.67 (-2.47-63.82), p=0.07 |

^1^Adjusted estimates, accounting for cluster-level variation using mixed effects linear regression and

adjusting for covariates (caste, delivery attendant, gravidity, mother’s age, mother’s education,

baby’s sex, multiple births).

^2^Crude estimates, accounting for cluster-level variation using mixed effects linear regression.

**Supplemental Table 2**. Morbidity of infants in the neonatal period in intervention and

comparison clusters.

| **Morbidity Outcomes** | **Total**  **N** | **No**  **N** | **Yes**  **N (% of total)** | **Odds ratio^1^** | **p-value^1^** |
| --- | --- | --- | --- | --- | --- |
| **Hospitalization^2^** | | | | | |
| ***Intention-to-treat*** | | | | | |
| Intervention | 13,478 | 13139 | 339 (2.5) | 0.93 (0.73, 1.19) | 0.575 |
| Comparison | 13,109 | 12789 | 320 (2.4) |  |  |
| ***Per protocol*** | | | | | |
| Exclusive sunflower seed oil (SSO) in Intervention | 4,096 | 4021 | 75 (1.8) | 0.58 (0.41, 0.82) | **0.002*** |
| Exclusive mustard oil (MO) in Comparison | 4,720 | 4584 | 136 (2.9) |  |  |
| **Any illness^3^** | | | | | |
| ***Intention-to-treat*** | | | | | |
| Intervention | 12,449 | 11103 | 1346 (10.8) | 0.84 (0.67, 1.07) | 0.161 |
| Comparison | 12,180 | 10657 | 1523 (12.5) |  |  |
| ***Per protocol*** | | | | | |
| Exclusive SSO in Intervention | 3,838 | 3576 | 262 (6.8) | 0.56 (0.41, 0.77) | **<0.001*** |
| Exclusive MO in Comparison | 4,222 | 3781 | 441 (10.4) |  |  |
| **Skin infection^4^** | | | | | |
| ***Intention-to-treat*** | | | | | |
| Intervention | 13,478 | 12966 | 512 (3.8) | 1.19 (0.84, 1.69) | 0.334 |
| Comparison | 13,109 | 12630 | 479 (3.6) |  |  |
| ***Per protocol*** | | | | | |
| Exclusive SSO in Intervention | 4096 | 3987 | 109 (2.7) | 0.90 (0.55, 1.48) | 0.679 |
| Exclusive MO in Comparison | 4,720 | 4553 | 167 (3.5) |  |  |
| **Umbilical disorder^5^** | | | | | |
| ***Intention-to-treat*** | | | | | |
| Intervention | 12,443 | 11359 | 1084 (8.7) | 1.02 (0.73, 1.42) | 0.908 |
| Comparison | 12,188 | 11146 | 1042 (8.5) |  |  |
| ***Per protocol*** | | | | | |
| Exclusive SSO in Intervention | 3,832 | 3600 | 232 (6.1) | 0.69 (0.45, 1.06) | 0.092 |
| Exclusive MO in Comparison | 4,218 | 3914 | 304 (7.2) |  |  |

^1^For intention-to-treat models, adjusted estimates are shown from individual-level mixed-effects

logistic regression analysis accounting for within-cluster variations and adjusting for covariates

(caste, first-visit weight, delivery attendant, gravidity, maternal age, maternal education,

sex of the baby, and multiple births). For per protocol models, crude estimate are shown from

individual-level mixed-effects logistic regression analysis accounting for within-cluster variations.

^2^“Was the baby ever hospitalized?”

^3^“Did the baby ever suffer from any health problem?”

^4^“Did the baby ever have any boil on the skin with pus in it?”

^5^“Did the baby ever suffer from a cord problem?”
